# The world has gone mad

**DOI:** 10.1017/ehs.2023.32

**Published:** 2023-12-11

**Authors:** Ruth Mace

**Affiliations:** University College London, London WC1H 0PT, UK

## Abstract

**Figure:**
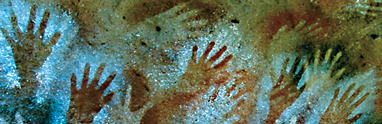

Horror and grief has been streamed into our TVs and phones for many weeks now. So total is the coverage of Gaza it has crowded out most other news, despite much else in need of coverage happening around the world. One might be tempted to think – it is not in my country and I cannot influence anything, so I can ignore it. However, emotions are powerful things, evolved for good reason; so nearly everyone finds themselves affected at some level by seeing such loss of life. It is hard to write anything light-hearted about this last year, as it draws to its end.

On the subject of sensitive topics, we have a special collection underway on researching sensitive topics, edited by Olympia Campbell and myself. We will provide examples of how to try and research things that people find hard to speak about openly or truthfully. Obviously only some sensitive topics will be in there; some topics are really not worth researching. Yet there are many difficult topics in our field that we do not want to go un-investigated, so we will do our best to try and help you study them.

We also have our special collection on causal inference, edited by Charles Efferson, Joseph Bulbulia and James Holland Jones, which I know you eagerly await. A few papers are out (e.g. Bromham & Yaxley, 2023; Major-Smith, 2023) and many more on the verge of publication; I can assure you it is nearly there. We are also just starting to compile a special collection on cultural evolution of the arts, guest edited by Oleg Sobchug and Mason Youngblood. I really look forward to all these collections coming to fruition. If you would like to guest edit your own special collection, on topics great or small, please do get in touch.

Thank you all, as always, for your support for *Evolutionary Human Sciences* over the last year. It is worth noting we have now completed our fifth volume. Time flies. I am not religious, but ritual is another thing altogether, and the Christmas ritual starts with turning on the Aga. If you have never encountered an Aga, it is a big, old, oven that has to be left on continuously, that used to be popular with middle class Brits. It is wonderful for warming your kitchen, baking cakes and roasting meat. Unfortunately it is also a crime against global warming, so ours is now turned off for 51 weeks of the year. However, for Christmas week we turn it back on. Have a very happy holiday, and let's wish, more than ever, for a peaceful New Year.
